# Long-Term Effects of Short-Term Music Therapy for Prison Inmates: Six-Year Follow-Up of a Randomized Controlled Trial

**DOI:** 10.1177/0306624X20909216

**Published:** 2020-03-13

**Authors:** Christian Gold, Fredrik B. Due, Elin K. Thieu, Kjetil Hjørnevik, Lars Tuastad, Jörg Assmus

**Affiliations:** 1 GAMUT – The Grieg Academy Music Therapy Research Centre, NORCE Norwegian Research Centre, Bergen, Norway; 2 Grieg Academy – Department of Music, University of Bergen, Norway; 3 Bjørgvin Prison, Breistein, Norway

**Keywords:** music therapy, offenders, psychosocial interventions, randomized controlled trial, recidivism, relapse prevention

## Abstract

For most interventions to reduce criminal recidivism, long-term effects are uncertain. Music therapy has shown effects on possible precursors of recidivism, but direct evidence on long-term effects is lacking. In an exploratory parallel randomized controlled trial, 66 inmates in a Norwegian prison were allocated to music therapy or standard care and followed up over a median of 6 years, using state registry data. Median time to relapse was 5 years, with no differences between the interventions. The imprisonment of most participants was too short to provide a sufficient number of therapy sessions. Sufficiently powered studies are needed to examine the long-term effects of appropriate doses of therapy.

## Background

Criminal recidivism refers to relapse into criminal behaviour and is a worldwide problem. Internationally, recidivism rates of prisoners have been reported to be as high as 50%; however, they vary considerably not only between countries, but also depending on sample characteristics/types of offences and definitions of recidivism (Fazel & Wolf, 2015). Two-year conviction rates were the most commonly reported outcome across 11 countries. Such reconviction rates ranged from 20% in Norway through around 30% in other Scandinavian countries to around 50% in many other countries across Europe and North America (Fazel & Wolf, 2015). Nordic countries in general have a “reputation for . . . low recidivism” (Fazel & Wolf, 2015, p. 6). Prisoners are also at high risk of mental health problems (Fazel & Danesh, 2002).

Few interventions for prisoners have been successfully tested in randomized controlled trials (RCTs; Farrington & Welsh, 2005). The difficulty of conducting RCTs in this area has been generally acknowledged and has been attributed to a variety of reasons, ranging from difficulties with voluntary informed consent to biases in self-reports, in a population that is especially vulnerable and has a relatively monotonous life (National Institute of Justice, 2012). Therefore, many researchers have relied on weaker quasi-experimental designs. In the few cases where RCTs have been used, they have sometimes led to surprising results. For example, one RCT found an unintended detrimental effect of higher prison security levels on returning to prison during 6 years after release; this finding was in contradiction to ideas of deterrence, but suggested a role of peer group, inmate culture, and environmental strain (Gaes & Camp, 2009). RCTs of specific populations of offenders with additional health-related problems (e.g., mental health problems or drug abuse) are more common; however, many of those RCTs also found no effects of interventions on reoffending, rearrest, or reincarceration (Dennis et al., 2012; Khan et al., 2015; Livingstone et al., 2013; Perry et al., 2019; Perry, Neilson, Martyn-St James, Glanville, Woodhouse, et al., 2015; Perry, Neilson, Martyn-St James, Glanville, Woodhouse, & Hewitt, 2015).

Music therapy in prison was first described by Wardle (1979), who explored the use of music therapy techniques with women living in a psychiatric unit in prison. A majority of the studies following the next decades focused on music therapy either as a treatment or as an agent for behavioural change (Cohen, 1987; Daveson & Edwards, 2001; Fulford, 2002; Gallagher & Steele, 2002; Glyn, 2003; Hakvoort, 2002; Hoskyns, 1995; Loth, 1994; Reed, 2002; Smeijsters & Cleven, 2006; Thaut, 1987). More recently, O’Grady (2011) conducted a case study of her work with a theatre company inside a maximum-security women’s prison in Australia, focusing on the potentials of performance related to aspects of individual development, well-being, and relational and community levels of change. In a mixed-methods study of music therapy with women in a U.K. prison, Leith (2014) showed that prisoners attending music therapy experienced a positive change in self-perception. In an RCT of 200 male prisoners, Chen, Hannibal, and Gold (2016) found music therapy to improve anxiety, depression, and self-esteem. In the context of music therapy in Norway, several qualitative studies have scrutinized the national rehabilitation project “Music in Custody and Liberty” which draws upon a community music therapy approach (Finsås & Tuastad, 2008; Mortensen, 2006; Nilsen, 1996; Tuastad, 2014; Tuastad & O’Grady, 2013). In an ethnographic study of musical life in a low-security prison in Norway, Hjørnevik and Waage (2019) describe musicking as an everyday practice in prison and explore how musicking forms the prisoners’ emotional life.

In summary, goals of music therapy with offenders have primarily been related to improving mental health and/or facilitating behavioural change (Coutinho et al., 2015). Community-oriented approaches have also emphasized the wider social and cultural needs of clients in processes of reintegration to society (Leith, 2014; Tuastad & Stige, 2015). Music therapy has been shown to improve the mental health of offenders; however, its longer-term effects on recidivism are unknown (Chen, Leith, et al., 2016). The aim of this study was to examine the long-term effects on criminal recidivism in a 6-year follow-up study of a pilot RCT of the effects of music therapy for prison inmates (Gold et al., 2014).

## Method

### Design and Setting

In a single-centre parallel exploratory pragmatic RCT at Bjørgvin Prison near Bergen, Norway, inmates who consented to participate were randomized to either music therapy or standard activities in 2008 to 2009 (Gold et al., 2014). The newly established “model prison” aimed to establish new interventions that might help in the rehabilitation process. In dialogue with the prison staff and affiliated researchers, a pragmatic design with broad inclusion criteria and flexible interventions was chosen, because neither the prospective population nor the best working modalities for this population were known precisely. The trial was powered to determine short-term mental health outcomes; no effects were found on these outcomes; however, follow-up rates were low (Gold et al., 2014). The trial also included physiological short-term outcomes (Gold & Assmus, 2015) as well as a plan for a longer-term follow-up of recidivism outcomes based on state registry data (http://www.isrctn.com/ISRCTN22518605), which are presented here. The original study as well as this follow-up study were approved by the Regional Committee for Medical and Health Research Ethics Western Norway (REK Vest) and conducted in accordance with the relevant guidelines and regulations. The trial is registered in the ISTCRN Register (No. ISRCTN22518605).

### Participants

All prisoners who had sufficient command of the Norwegian language and provided written informed consent to participate in the study, as well as separate written consent to the retrieval of data from official criminal records on any possible new conviction or writ within a 10-year period after inclusion in the study, were eligible to participate. All prisoners were convicted of a crime (i.e., not awaiting trial); crime types included violence, sexual offences, drug-related offences, theft/robbery, and fraud. Sentence durations varied from a few days to several years, with an average of about 60 days (Gold et al., 2014).

### Interventions

After randomization, one male music therapist, who was employed in the prison for the purpose of the study, invited participants randomized to music therapy to participate in sessions. The therapist adhered to a client-centred and resource-oriented approach to music therapy (Hjørnevik & Waage, 2019; Rolvsjord et al., 2005), involving a focus on the clients’ motivations, needs, and agency in codetermining music therapeutic aims and associated musical activities for the sessions. In line with the therapist’s approach and the pragmatic design of the trial, music therapy was offered flexibly in terms of format (usually group, but in some cases individual), frequency of sessions (typically 2–3/week), duration of each session, and music therapeutic methods and techniques. The music therapist decided the length and frequency of sessions based on client interests combined with an evaluation of what frequency would be appropriate to (a) meet the music therapeutic aims, (b) not exert excessive pressures on the individual, and (c) not cause saturation of the therapeutic relationship. The final number of recorded frequencies was also affected by any cancellations or irregularities, for example, due to sickness or unforeseen parole for the client. Activities included playing in bands, instrumental tuition, recording music, music improvisation, songwriting, and verbal reflections of the music experiences (Gold et al., 2014). Many of these activities were offered in combination or in sequence within one session and were selected on the basis of client preferences combined with the music therapist’s evaluation of which activities would be optimal in (a) meeting the music therapeutic aims and (b) meeting the perceived ongoing or immediate mental health needs of the clients, for example, affect regulation or alleviating symptoms of anxiety and/or depression. These evaluations were based on the presentation of the client within sessions, the development of the therapeutic relationship through ongoing verbal and musical interactions, and, in group settings, unfolding group dynamics. Standard activities offered at the prison included work- and school-related rehabilitative activities.

### Outcomes

Registry data were obtained from Statistics Norway (Statistisk sentralbyrå [SSB]). This database contains official statistics, which are derived from police records and the Norwegian National Collection Agency (Statens innkrevingssentral). We sought data on convictions and writs of each participant in the time from enrolment in the trial to the end of 2014, which at the time of inquiry was the last date until when statistics at SSB were complete. Data received from SSB included dates on all new crimes committed in the participant’s individual time periods as well as the type of sentence given (Table 1). We also applied for registry data from the National Police Directorate of Norway (Politidirektoratet [POD]), but this was not successful. The data obtained from SSB were transformed into time-to-event data, where only the date of the first occurring crime for each participant was included, using the date of the criminal event (not the date of conviction). Criminal events were classified into two types of events: any events (including writs) and serious events (all other events listed in Table 1, excluding writs).

**Table 1.** Types of Events Analysed in This Study.

Norwegian definition	English translation	Explanation
Ubetinget fengsel alene, inkludert militær arrest	Prison sentence (not suspended), including military imprisonment	The given prison sentence shall be carried out, as soon as there is a prison available for the convicted person. This also includes military imprisonment
Ubetinget og betinget fengsel, inkludert ubetinget + betinget + bot	Prison sentence and suspended prison sentence, included prison sentence + suspended sentence + fine	
Ubetinget fengsel og annen, inkludert ubetinget + bot	Prison sentence and others, including prison sentence + fine	
Forelegg alene	Writ	Giving the option of a fine or confiscation or both in lieu of prosecution
Samfunnsstraff alene	Community sentence	Sentenced to execute a certain amount of hours duties of public utility. Milder than prison sentence, often given to younger offenders, and as rehabilitation for substance abuse
Samfunnsstraff og annen, inkludert kode 57 og 58, dvs samfunnsstraff er dominant reaksjon over betinget fengsel	Community sentence and other, where community sentence is given as the dominant sentence	
Betinget fengsel	Suspended prison sentence	The prison sentence is suspended and is not carried out if certain terms are fulfilled within the given period, for example, not committing new crimes
Betinget fengsel og bot	Suspended prison sentence and fine	
Tvungent psykisk helsevern	Compulsory psychiatric care	Involuntary commitment or civil commitment (also known as sectioning in some jurisdictions) is a legal process through which an individual who is deemed by a qualified agent to have symptoms of severe mental disorder is court-ordered into treatment in a psychiatric hospital (inpatient) or in the community (outpatient)

*Note*. Codes and Norwegian definitions as provided by Statistisk sentralbyrå (SSB); translations by the authors according to Bø (2007).

### Data Analysis

Data were analysed on an intention-to-treat basis, that is, all those who were randomized were analysed, regardless of whether or not they received the intervention as intended. We used a two-sided significance level of .05. We used Kaplan–Meier curves to analyse the time from randomization to a new criminal event. As proportional hazards could not be assumed, interventions were compared using Breslow tests. As serious events are a subgroup of all events, we did not adjust for multiple testing. Computations were performed in R 3.4 (www.r-project.org) with the package survMisc 0.5.4; graphical analyses were created in R and MATLAB 9.0 (The MathWorks, Inc., Natick, MA). We had also planned per-protocol analyses for those who had received at least 10 or 20 sessions, but these analyses were not possible because the size of this subsample was too small. For the same reason, we were unable to test associations between baseline variables and recidivism.

## Results

### Participants Included in the Study

**Figure 1.**
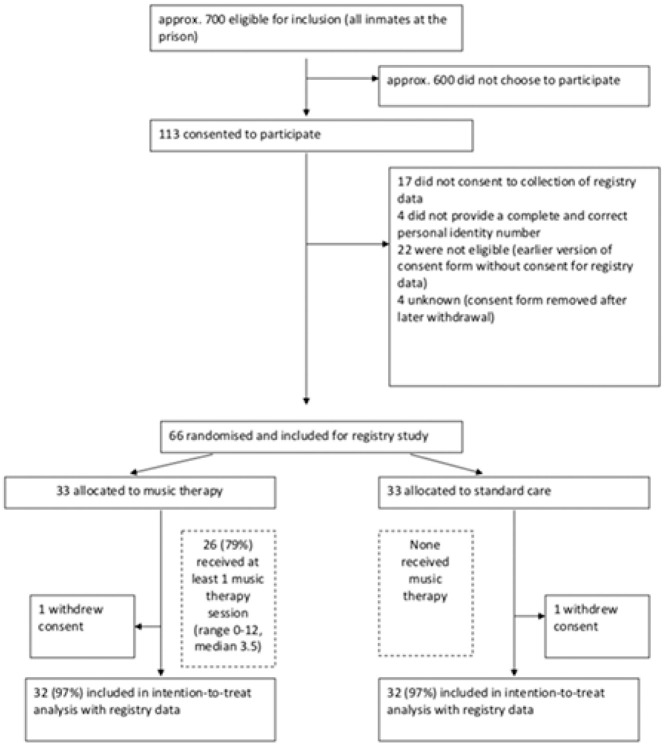
Flow of participants through the study.

Of 113 who consented to participate in the original trial (Gold et al., 2014), only 66 had agreed to the registry-based follow-up study and had provided their personal number (Figure 1). Thus, the intention-to-treat sample for this study consisted of 66 participants, of whom 33 were allocated to music therapy and 33 to standard care. Two participants, one from each arm, withdrew after randomization. Thus, the outcome data were available for 64 (97%) of the 66 who were initially included. All 64 participants included in this follow-up study were followed up until the end of 2014; the median time of follow-up was 6.1 years (range = 5.5–6.5 years). Baseline characteristics of the participants are shown in Table 2.

**Table 2.** Baseline Characteristics of Study Participants.

Baseline characteristic	All participants (*N* = 64)	Standard care (*n* = 32)	Music therapy (*n* = 32)	*p*
Number of therapy sessions, median [range]	0 [0–12]	0 [0–0]	3.5 [0–12]	—
Age (years)^a^, median [range]	26 [18–53]	25 [19–53]	28 [18–52]	.164
Expected stay (days)^a^, median [range]	32 [7–454]	33 [7–454]	27 [10–329]	.700
State anxiety (STAI-state)^b^, *M* (*SD*)	39.3 (13.1)	40.8 (12)	37.7 (14)	.346
Trait anxiety (STAI-trait)^b^, *M* (*SD*)	43.6 (11.1)	45.8 (10.5)	41.4 (11.4)	.115
Anxiety (HADS-A)^b^, *M* (*SD*)	7.4 (4.5)	7.9 (4.2)	6.9 (4.9)	.379
Depression (HADS-D)^b^, *M* (*SD*)	5.9 (3.9)	5.8 (3.4)	5.9 (4.5)	.901
Social relationship (Q-LES-Q)^b^, *M* (*SD*)	40.2 (7.5)	39.7 (6.8)	40.7 (8.2)	.596
Anxiety above cutoff (HADS-A ≥ 8)^c^, *n* (%)	26 (40.6)	15 (46.9)	11 (34.4)	.505
Depression above cutoff (HADS-D ≥ 8)^c^, *n* (%)	21 (32.8)	11 (34.4)	10 (31.2)	1.000
RSQ.2.anx^b^, *M* (*SD*)	2.3 (0.9)	2.2 (1)	2.3 (0.9)	.794
RSQ.2.avo^b^, *M* (*SD*)	2.6 (0.6)	2.6 (0.6)	2.6 (0.7)	.959
RSQ.4.sec^b^, *M* (*SD*)	3.2 (0.6)	3.2 (0.5)	3.3 (0.7)	.784
RSQ.4.fea^b^, *M* (*SD*)	2.8 (0.8)	2.9 (0.7)	2.8 (0.8)	.546
RSQ.4.prc^b^, *M* (*SD*)	2.6 (0.8)	2.5 (0.8)	2.7 (0.8)	.403
RSQ.4.dsm^b^, *M* (*SD*)	3.3 (0.6)	3.3 (0.5)	3.3 (0.7)	.876

*Note*. STAI = State–Trait Anxiety Inventory; HADS = Hospital Anxiety and Depression Scale; Q-LES-Q = Quality of Life Enjoyment and Satisfaction Questionnaire; RSQ = Relationship Scale Questionnaire; for details of scales, see Gold et al. (2014).

a Mann–Whitney *U* test. ^b^*t* test. ^c^χ^2^ test.

### Interventions Received

Due to short sentences, most participants did not receive the full intervention (Gold et al., 2014). Of those randomized to music therapy, six (18%) did not receive any music therapy; the mean number of sessions was 4.35 (*SD* = 3.89; median = 3; maximum = 12).

### Recidivism Over Time

**Figure 2.**
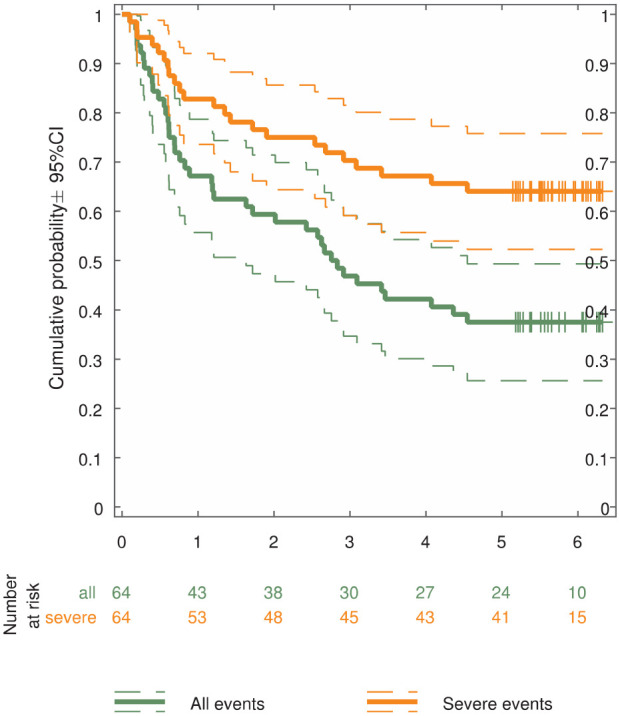
Kaplan–Meier curves of probability for criminal relapse in 64 Norwegian prisoners—all versus serious events. *Note*. This figure shows the survival outside prison over 6 years after release for two types of recidivism (green: any event in the criminal record, including writs; orange: only more serious events, excluding writs). Dashed lines indicate 95% confidence intervals. Vertical lines indicate censoring (i.e., shorter follow-up due to later inclusion).

Across the sample, about 20% had *serious* recidivism events during the first year and about 25% during the first 2 years, with a flattening curve after that (Figure 2). When including *all* events, the risk was about one third in the first year, reaching 50% after 32 months and flattening over time. After 5 years, about one third had had a serious recidivism event, another third had had a nonserious recidivism event, and the remaining third had had no recidivism event.

### Effects of Music Therapy on Recidivism

**Figure 3.**
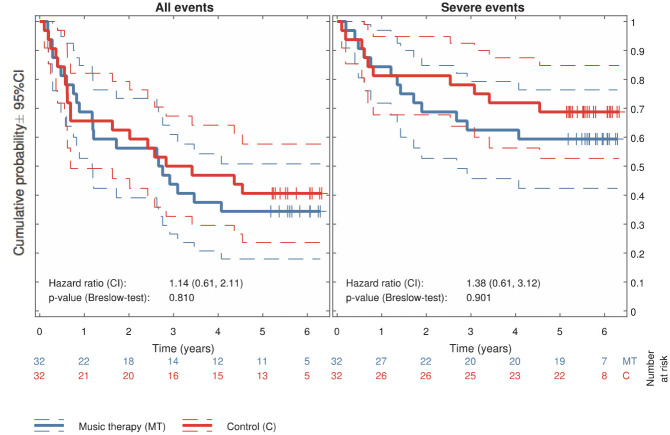
Kaplan–Meier curves of probability for criminal relapse in 64 Norwegian prisoners randomized to music therapy or control. *Note*. This figure shows the survival outside prison over 6 years after release for participants randomized to music therapy (blue) or control (red). Left panel: any event in the criminal record, including writs; right panel: only more serious events, excluding writs. Dashed lines indicate 95% confidence intervals. Vertical lines indicate censoring (i.e., shorter follow-up due to later inclusion). CI = confidence interval.

No difference was found between music therapy and standard care with respect to either all events or serious events (Figure 3). For serious events, the hazard ratio was 1.38 (95% confidence interval [CI] = [0.61, 3.12]; *p* = .901). For all events, the hazard ratio was 1.14 (95% CI = [0.61, 2.11]; *p* = .810).

## Discussion

### Findings of This Study in the Context of Developing Knowledge on Recidivism

This was, to our knowledge, the first randomized long-term follow-up of music therapy for prisoners. As discussed in the initial study report of short-term outcomes (Gold et al., 2014), as well as our subsequent meta-analysis (Chen, Leith, et al., 2016), the very low number of music therapy sessions provided, due to the short sentence of many participants, limited the ability of this study to find any effects. It is therefore not surprising that no significant effects of short-term music therapy on long-term recidivism were found in this follow-up study. However, CIs were wide and did not exclude potentially meaningful effects. A replication would therefore be warranted and should consider the following.

The population in our study had varying, but mostly very short sentences. This influenced the extent to which the intervention could be provided. It may also have influenced the event rates, which were lower than we expected. We found that 25% of all participants had a serious recidivism event (i.e., any event excluding writs) during the first 2 years after release. Comparing recidivism rates directly between studies is difficult due to variations between samples and outcome definitions. The review by Fazel and Wolf (2015) compared recidivism data for 18 countries, but noted that although definitions of “recidivism” varied widely, 2-year “reconviction” rates were most commonly reported. Table 3 in their study compares this outcome (2-year reconviction rates) across 11 countries, finding 20% in Norway but 27% to 59% in other countries. Nordic countries were analysed separately because of their “reputation for . . . low recidivism” (Fazel and Wolf, 2015, p. 6). However, even “in Norway, 2-year recidivism rates ranged from 14%-42%,” depending on the definition of the sample and outcome (Fazel and Wolf, 2015, p. 6). Thus, the rate found in this study appears to be low compared with typical recidivism rates reported elsewhere (Fazel and Wolf, 2015; Kristofferson, 2013). This may be explained by a focus of Norwegian prisons on reintegration (Kriminalomsorgen, 2018), but also by general societal aspects such as a good social safety net (Deady, 2014).

### Limitations of the Study

Due to the inevitable delay from crime to conviction, we may have missed some events. Conversely, a strength of this methodology is the ability for complete follow-up, which is not biased by differential dropout in response to treatment. Another important strength is the objectivity of these data: In contrast to many other measures, detection bias is not a problem in this study design.

Retrospectively, one may wonder if the lack of short-term effects reported previously (Gold et al., 2014) may be indicative of a likely lack of long-term effects as well. However, there are examples of interventions that have delayed effects and this may also be true for music therapy. Furthermore, recidivism is different from the mental health outcomes examined previously. In spite of the clear link between poor mental health and imprisonment, interventions may improve recidivism without improving mental health symptoms. Alternatively, it may be that existing effects on mental health were not detectable; as noted above, a strength of this study was its complete and objective follow-up. Independently from those considerations, it is imperative in terms of the replicability of research that all preplanned outcomes from all RCTs are reported, also if they are negative (see, e.g., the AllTrials initiative in medicine; www.alltrials.net).

A further limitation of the study is that it was not designed to investigate any specific mechanisms of change. This is in line with the pragmatic trial design, where the goal is to help “choose between options of care” rather than to “test causal hypotheses” (Thorpe et al., 2009, p. 464). The normal care that all prisoners, including those in the control condition, received comprised participation in various work- and education-related activities. Participation in these activities would be expected to vary individually, for example, as a function of an individual’s passion or sense of commitment. However, due to the randomized trial design, any standard activities would be expected to be similarly distributed between the groups, so individual differences in commitment to those activities would average out across groups. In contrast, music therapy was only offered to one group, again selected randomly from the whole sample, thus precluding any bias due to interindividual differences in commitment. In other words, both groups were likely to contain participants with high and low commitment.

However, a passion to pursue something or a sense of commitment may act as an underlying mechanism for both the standard activities and the music therapy offered at the prison. Our sample was diverse with regard to the participants’ prior interest in or experience with music. Some participants had a strong interest in music, an identity as hobby musicians, or a motivation to develop their musical skills, whereas other participants had less of an a priori motivation or interest. The study was advertised as “participation in a research project” rather than “participation in music therapy” to avoid introducing a bias towards musically interested, skilled, or experienced participants. Whether prisoners in general have a relatively high interest in music would have to be investigated (Gold et al., 2013). Qualitative research suggests that a high commitment and sense of responsibility towards one’s band may be a key mechanism of music therapy to prevent new criminal activity among former prisoners (Tuastad & Stige, 2015).

### Implications for Future Research

This follow-up study has shown that a long-term follow-up of an RCT in Norwegian prisoners, using complete and objective state registry data of criminal recidivism, is feasible. Future studies should attempt to estimate the required sample based on expected sentence length, number of therapy sessions, and event rates, and where possible be complemented by qualitative accounts to deepen our understanding of the relationships between therapeutic mechanisms, therapy outcomes, and research results in this field. Based on studies of music therapy in related areas, we would suggest that inclusion criteria specifying a sentence length of minimum 3 months and the corresponding durations of (weekly or biweekly) interventions would be meaningful. The effects of music therapy for people with serious mental disorders have been found to increase with the number of sessions (Gold et al., 2009), and the same is likely with prisoners (Chen, Leith, et al., 2016). Based on recent qualitative research in the field (Tuastad, 2014), studies measuring recidivism should also involve music therapy in the community after release. Although flexibility in the use of music therapy techniques is necessary to meet individual needs, the interventions offered should be uniform across the sample in relation to frequency, session length, and basic structure to ensure sufficient grounds for comparison.

Large sample sizes will be necessary to detect meaningful effects reliably. For example, to achieve 80% power in a two-sided test of proportions, and assuming a 30% recidivism risk in the control group over 5 years, 120 participants per arm are needed to detect a relative risk reduction of 50%. Alternatively, if only a 20% relative risk reduction is expected, more than 800 participants per arm would be required to achieve the same test power.^1^

Finally, music therapy is rarely targeted at one single outcome. However, preventing recidivism is clearly one of the most relevant long-term, “downstream” outcomes to address in this population. Continuity of interventions, also beyond release from prison, will likely be important to achieve this goal.

## Notes

1. Power analysis conducted in R: power.prop.test(p1=0.30, p2=0.15, power=0.80); power.prop.test(p1=0.30, p2=0.24, power=0.80).
